# Bacteriophage evolution drives *Pseudomonas aeruginosa* PAO1 biofilm diversification

**DOI:** 10.1186/1471-2105-12-S11-A2

**Published:** 2011-11-21

**Authors:** Kerensa McElroy, Fabio Luciani, Janice Hui, Scott Rice, Torsten Thomas

**Affiliations:** 1 Centre for Marine Bioinnovation, UNSW, Sydney, NSW 2052, Australia; 2 Inflammatory Diseases Research Unit, UNSW, Sydney, NSW 2052, Australia

## Background

Pseudomonas aeruginosa infection is the leading cause of death for Cystic Fibrosis patients. Antibiotic resistance is rife, possibly due to high colonising population diversity. Our lab has replicated phenotypic diversification in a P. aeruginosa PAO1 biofilm model of lung infection. To reveal underlying genetic variants, we deep-sequenced PAO1 biofilm samples. Our analysis demonstrates several techniques for extracting meaningful biological information from error-prone sequencing data.

## Materials and methods

DNA was extracted from PAO1 biofilm samples harvested after four and 11 days of growth, and Illumina sequenced to >1000x coverage. Sequencing was also simulated from computer generated PAO1 haplotypes with our program GemSIM. GemSIM uses real data (e.g., the PhiX control) to generate run-specific error models, facilitating realistic simulation. After optimisation, the program VarScan {Koboldt, 2009 #48} accurately detected SNPs in simulated data with frequencies down to 5%. VarScan was then used to identify SNPs with frequency >=5% in the biofilm data.

In high diversity regions, haplotypes were reconstructed using bayesian statistical techniques implemented in the program ShoRAH {Zagordi, 2010 #49}, and validated through analysis of individual reads. (ShoRAH’s error-correction algorithm can accurately identify variants in high diversity areas with frequencies down to 0.1%.)

## Results and conclusions

Surprisingly, the PAO1 genome contained only two SNPs, with frequencies around 10%. Both were within a large hypothetical outer-membrane protein postulated to be involved in biofilm formation and antibiotic resistance. Both SNPs were silent; however converted rare codons to more common ones, potentially increasing expression. These SNPs may reflect early biofilm lifestyle adaptation.

In contrast to the negligible genetic diversity of PAO1, its associated bacteriophage Pf4 revealed ongoing diversification, characterised by an increase in Shannon’s entropy between days four and 11 and an explosion in phage population size. All phage SNPs were within or upstream from the putative Repressor C gene. This gene is implicated in Pf4 superinfectivity, which results in loss of host resistance and conversion from a lysogenic form to a lethal, lytic lifecycle.

**Figure 1:**
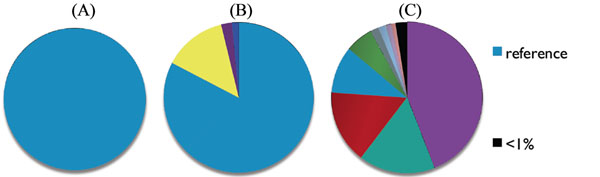
Relative phage haplotype frequencies in PAO1 samples, (A) planktonic, (B) biofilm 4 days, (C) biofilm 11 days.

In total, nine Pf4 haplotypes with frequencies > 1% emerged by day 11, while the original haplotype dropped to 9% (Fig. 1). These results suggest superinfective phage haplotype emergence drives diversification within PAO1 biofilms.
